# High quality draft genome sequence of *Meganema perideroedes* str. Gr1^T^ and a proposal for its reclassification to the family *Meganemaceae* fam. nov.

**DOI:** 10.1186/s40793-015-0013-1

**Published:** 2015-02-27

**Authors:** Simon J McIlroy, Alla Lapidus, Trine R Thomsen, James Han, Matthew Haynes, Elizabeth Lobos, Marcel Huntemann, Amrita Pati, Natalia N Ivanova, Victor Markowitz, Susanne Verbarg, Tanja Woyke, Hans-Peter Klenk, Nikos Kyrpides, Per H Nielsen

**Affiliations:** Department of Chemistry and Bioscience, Centre for Microbial Communities, Aalborg University, Aalborg, Denmark; Theodosius Dobzhansky Center for Genome Bionformatics, St. Petersburg State University, St. Petersburg, Russia; Algorithmic Biology Lab, St. Petersburg Academic University, St. Petersburg, Russia; DOE Joint Genome Institute, Walnut Creek, California USA; Biological Data Management and Technology Center, Lawrence Berkeley National Laboratory, Berkeley, California USA; DSMZ - German Collection of Microorganisms and Cell Cultures GmbH, Braunschweig, Germany; School of Biology, Newcastle University, Newcastle upon Tyne, United Kingdom; Department of Biological Sciences, King Abdulaziz University, Jeddah, Saudi Arabia

**Keywords:** Activated sludge, Bulking, Facultative methylotroph, Filamentous, *Meganema*, *Meganemaceae*, Wastewater

## Abstract

*Meganema perideroedes* Gr1^T^ is a filamentous bacterium isolated from an activated sludge wastewater treatment plant where it is implicated in poor sludge settleability (bulking). *M. perideroedes* is the sole described species of the genus *Meganema* and of the proposed novel family “*Meganemaceae”*. Here we describe the features of the type strain Gr1^T^ along with its annotated genome sequence. The 3,409,949 bp long draft genome consists of 22 scaffolds with 3,033 protein-coding and 59 RNA genes and is a part of *Genomic Encyclopedia of Type Strains*, Phase I: the one thousand microbial genomes KMG project. Notably, genome annotation indicated the potential for facultative methylotrophy. However, the ability to utilize methanol as a carbon source could not be empirically demonstrated for the type strain or for *in situ Meganema* spp. strains.

**Electronic supplementary material:**

The online version of this article (doi:10.1186/s40793-015-0013-1) contains supplementary material, which is available to authorized users.

## Introduction

**Figure 1:**
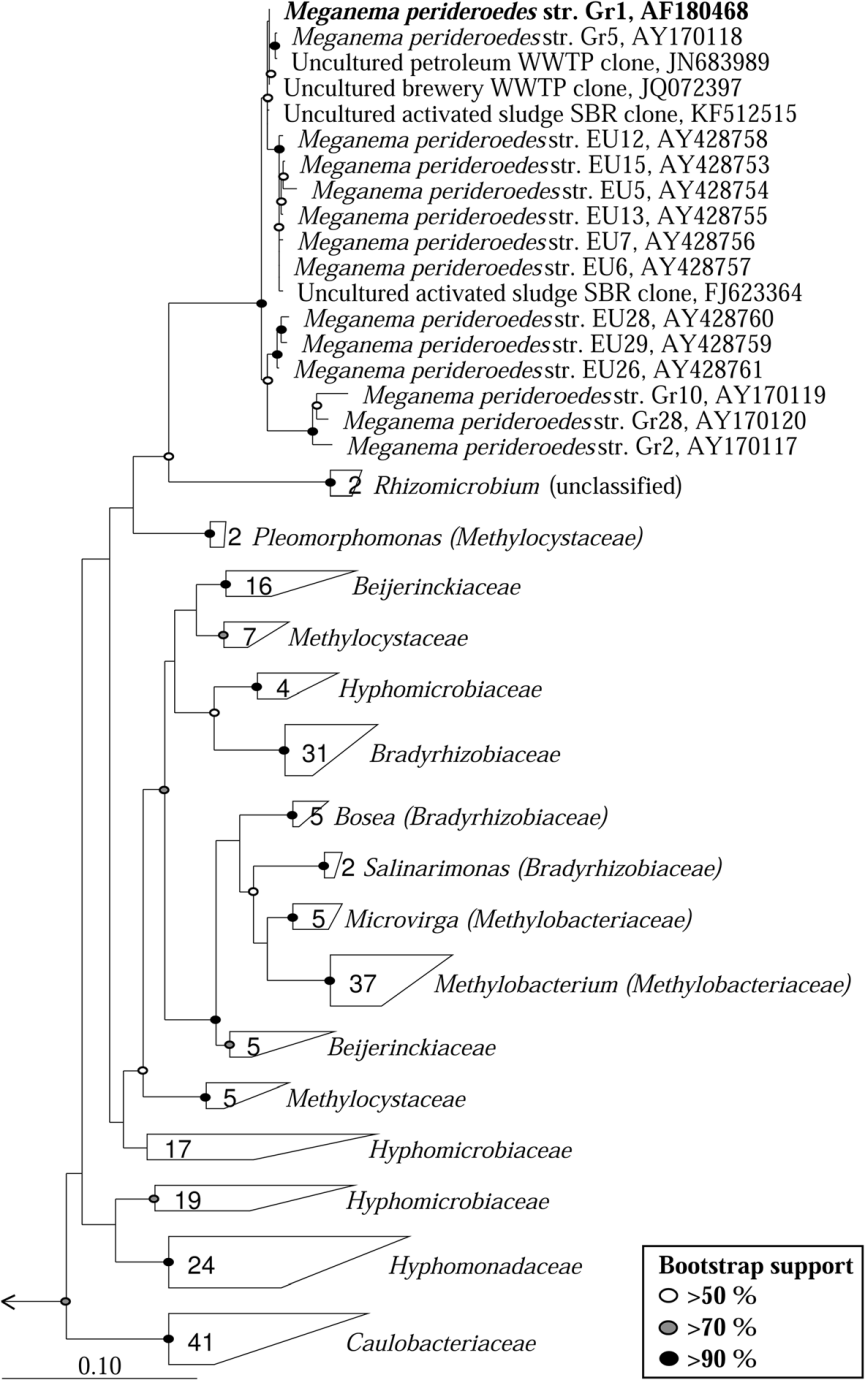
**Maximum-likelihood phylogenetic tree of 16S rRNA genes for all** ***Meganema*** **isolates and closely related species in the LTP database (LTPs111) [** 10 **] constructed using the ARB software [** 13 **].** The 1342 bp long sequence fragment of the unique 16S rRNA gene copy in the genome is identical with the previously published 16S rRNA gene sequence for the Gr1^T^ strain (AF18048). A 20% maximum frequency filter was applied in order to remove hypervariable positions. Included are all uncultured clone sequences from the NCBI database which share ≥94% sequence similarity with the Gr1^T^ strain (all were ≥ 98%), with sequences from the same study clustered at ≥ 99% similarity and a representative included. Bootstrap values, calculated from 100 re-samplings, are indicated for branches with > 50% support. Scale bar represents substitutions per nucleotide. The family *Fusobacteriaceae* was used as the out-group.

Strain Gr1^T^ (= DSM 15528 = ATCC BAA-740) is the type strain of *Meganema perideroedes* in the monospecific genus *Meganema* [1]. *M. perideroedes* is a filamentous bacterium isolated from an activated sludge WWTP in Denmark. All current isolates, along with 16S rRNA gene clone sequences in public databases, were isolated from activated sludge related sources (see Figure 1). High abundance of the filamentous form in these systems, though rarely reported, is associated with the sludge settleability problems known as bulking, and is therefore undesired [1,2]. *Meganema* spp. are often detected in lab-scale SBR systems optimized for PHA production for valuable bioplastics manufacture [3-8], and have a relatively high capacity for intracellular storage of such compounds [9], making them of potential biotechnological interest. Here we describe the features of the type strain Gr1^T^ along with its annotated genome sequence. The 3,409,949 bp long draft genome consists of 22 scaffolds with the 3,033 protein-coding and 59 RNA genes and is a part of *Genomic Encyclopedia of Type Strains*, Phase I: the one thousand microbial genomes (KMG) project.

## Classification and features

*M. perideroedes* Gr1^T^ was initially reported to be affiliated with the *Methylobacterium/Xanthobacter* group based on the common major fatty acid C_18:1_*ω*7*c* [1]*,* which presumably led to its later classification to the family *Methylobacteriaceae* in ‘The All-Species Living Tree Project Database’ (release LTPs111) [10]. However, in *The Prokaryotes Manual 4*^*th*^*Edition*, this classification was suggested to be erroneous [11]. The need for reclassification was primarily based on the lack of 16S rRNA gene relatedness of the *M. perideroedes* and other members of the *Methylobacteriaceae* family (see Figure 1), which was originally transcribed based solely on 16S rRNA based phylogeny [12]. Kelly and others [11] noted that *M. perideroedes* had no closely related species (none > 90% 16S rRNA gene similarity), with no phenotypic traits that specifically associate it with other described bacterial families. As such, the authors suggested that *Meganema* be classified as a novel family, designated “*Meganemaceae*”, within the order *Rhizobiales,* or alternatively transferred to the family *Caulobacteraceae* within the order *Caulobacterales* based on Greengenes taxonomy [13]. However, the latest release of the Greengenes taxonomy (October 2012) no longer classifies *Meganema* as such, and phylogenetic analysis does not appear to support its inclusion in either the *Methylobacteriaceae* or *Caulobacteriaceae* families (Figure 1). Therefore, we propose that the genus be classified to the novel family “*Meganemaceae*”*.*

**Figure 2:**
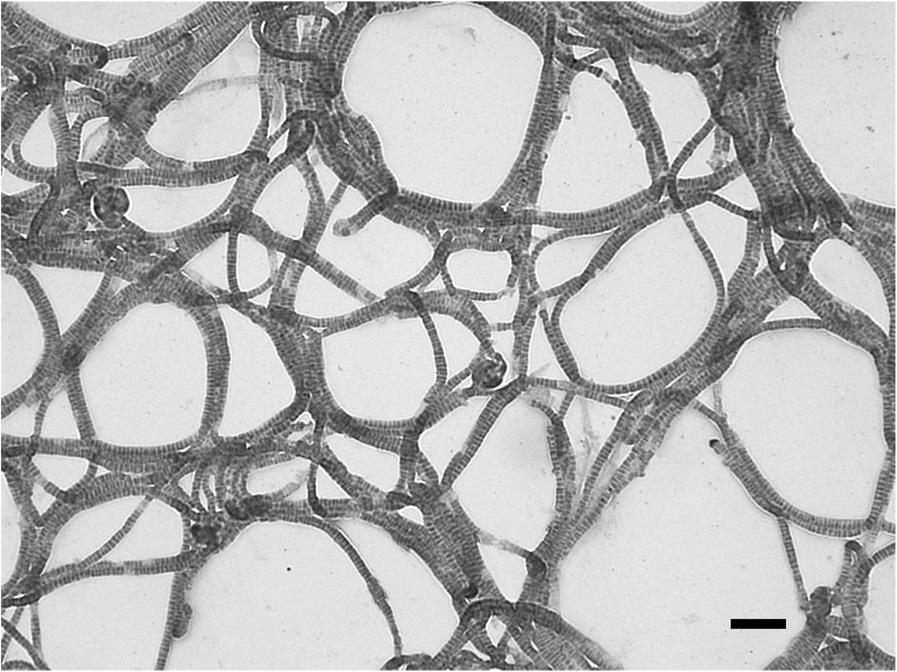
**Grey-scale brightfield micrograph of** ***M. perideroedes*** **Gr1** ^**T**^ **stained with safranin O.** Scale bar represents 10 *μ*m.

General features of *M. perideroedes* Gr1^T^ are summarized in Table 1. The strain exhibits a filamentous morphology with irregular disc shaped cells that are approximately 1.5-2 μm in diameter and Gram stain negative (Figure 2). They are non-motile and oxidase and catalase positive. Growth is observed in the presence of NaCl up to 2% [w/v] and between 15–35°C, with an optimum growth temperature of 25–30°C. In pure culture they produce off-white cohesive colonies that are difficult to separate. Cells are Nile Blue and Neisser stain positive, indicating intracellular lipid and polyphosphate inclusions, respectively. They have a demonstrated aerobic organoheterotrophic metabolism and are unable to utilize nitrate as an electron acceptor [1]. Starch or tributyrin are not hydrolysed. Carbon sources supporting growth of the Gr1^T^ isolate are unknown, although *in situ* strains of the genus were observed in activated sludge, with FISH-MAR, to assimilate acetate, propionate, butyrate, oleic acid, glucose, galactose, mannose, glycine and leucine, but not formate, pyruvate or ethanol [9].

**Table 1:** **Classification and general features of** ***M. perideroedes*** **Gr1** ^**T**^ [14]

**MIGS ID**	**Property**	**Term**	**Evidence code** ^**a**^
	Classification	Domain *Bacteria*	TAS [15]
		Phylum *Proteobacteria*	TAS [16]
		Class *Alphaproteobacteria*	TAS [17]
		Order *Rhizobiales*	TAS [18]
		Family “*Meganemaceae”*	TAS [11]
		Genus *Meganema*	TAS [1]
		Species *M. perideroedes*	TAS [1]
		Type strain Gr1	TAS [1]
	Gram stain	Negative	TAS [1]
	Cell shape	Irregular disc-shaped in filaments	TAS [1]
	Motility	Non-motile	TAS [1]
	Sporulation	Non-sporulating	NAS
	Temperature range	15-35°C	TAS [1]
	Optimum temperature	25-30°C	TAS [1]
	pH range; Optimum	Not reported	NAS
	Carbon source	Varied	NAS
MIGS-6	Habitat	Activated sludge	TAS [1,2,9]
MIGS-6.3	Salinity	Not reported	NAS
MIGS-22	Oxygen requirement	Aerobic	TAS [1]
MIGS-15	Biotic relationship	Free-living	TAS [1]
MIGS-14	Pathogenicity	Non-pathogen	NAS
MIGS-4	Geographic location	Grindsted, Denmark	TAS [1]
MIGS-5	Sample collection time	Not reported	NAS
MIGS-4.1	Latitude	55.758	NAS
MIGS-4.2	Longitude	8.924	NAS
MIGS-4.4	Altitude	41 m	NAS

^a^Evidence codes - IDA: Inferred from Direct Assay; TAS: Traceable Author Statement (i.e., a direct report exists in the literature); NAS: Non-traceable Author Statement (i.e., not directly observed for the living, isolated sample, but based on a generally accepted property for the species, or anecdotal evidence) [19].

The main respiratory quinone is Q-10 and the fatty acid profile is dominated by C_18:1_ω7*c* (86.4%) with smaller amounts of C_18:0_ (3.8%), C_16:0_ (2.9%), summed feature 2 (C_14:0_ 3-OH, C_16:1_ iso I)(2.4%), C_18:0_ 3-OH (2.3%) and C_19:0_ 10-methyl (1.1%) [1].

## Genome sequencing and annotation

### Genome project history

This organism was selected for sequencing on the basis of its phylogenetic position [20,21]. Sequencing strain Gr1^T^ (DSM 15528^T^) is part of the *Genomic Encyclopedia of Type Strains*, Phase I: the one thousand microbial genomes KMG project [22], a follow-up of the GEBA project [23], which aims to increase the sequencing coverage of key reference microbial genomes. The genome project is deposited in the Genomes OnLine Database [24] and the permanent draft genome sequence is deposited in GenBank. Sequencing, finishing and annotation were performed by the DOE JGI using state of the art sequencing technology [25]. A summary of the project information is shown in Table 2.

**Table 2:** **Project information**

**MIGS ID**	**Property**	**Term**
MIGS-31	Finishing quality	Level 2: High-Quality Draft
MIGS-28	Libraries used	Illumina Std. shotgun library
MIGS-29	Sequencing platforms	Illumina HiSeq 2000,
MIGS-31.2	Fold coverage	620.1 ×
MIGS-30	Assemblers	Velvet v. 1.1.04; ALLPATHS v. r41043
MIGS-32	Gene calling method	Prodigal
	Locus tag	B161DRAFT
	Genbank ID	ARFG00000000
	Genbank Date of Release	December 12, 2013
	GOLD ID	Gp0013029
	BIOPROJECT	169830
	Project relevance	Tree of Life, GEBA-KMG
MIGS-13	Source material identifier	DSM 15528

### Growth conditions and genomic DNA preparation

*M. perideroedes* Gr1^T^, DSM 15528, was grown in R2A medium (DSMZ medium 830) at 25°C [26]. DNA was isolated from 0.5-1.0 g of cell paste using Jetflex DNA purification kit (GENOMED 600100) following the standard protocol provided by the manufacturer but modified by an incubation time of 60 min, incubation on ice overnight on a shaker, the use of an additional 50 μl proteinase K, and the addition of 100 μl protein precipitation buffer. DNA is available through the DNA Bank Network [27].

### Genome sequencing and assembly

The draft genome sequence was generated using the Illumina technology [28]. An Illumina Standard shotgun library was constructed and sequenced using the Illumina HiSeq 2000 platform which generated 14,100,926 reads totaling 2,115.1 Mbp. All general aspects of library construction and sequencing performed at the JGI can be found at [29]. All raw Illumina sequence data was passed through DUK, a filtering program developed at JGI, which removes known Illumina sequencing and library preparation artifacts (Mingkun L, Copeland A, Han J. DUK. 2011, in preparation). The following steps were then performed for assembly: (1) filtered Illumina reads were assembled using Velvet [30], (2) 1–3 kbp simulated paired end reads were created from Velvet contigs using wgsim [31], (3) Illumina reads were assembled with simulated read pairs using Allpaths–LG [23]. Parameters for assembly steps were: 1) Velvet (velveth: 63 –shortPaired and velvetg: −very clean yes –export-Filtered yes –min contig lgth 500 –scaffolding no –cov cutoff 10) 2) wgsim (−e 0 –1 100 –2 100 –r 0 –R 0 –X 0) 3) Allpaths–LG (PrepareAllpathsInputs: PHRED 64 = 1 PLOIDY = 1 FRAG COVERAGE = 125 JUMP COVERAGE = 25 LONG JUMP COV = 50, RunAllpathsLG: THREADS = 8 RUN = std shredpairs TARGETS = standard VAPI WARN ONLY = True OVERWRITE = True). The final draft assembly contained 22 contigs in 22 scaffolds. The total size of the genome is 3.4 Mbp and the final assembly is based on 368.8 Mbp of Illumina data, which provides an average 108.2 × coverage of the genome.

### Genome annotation

Genes were identified using Prodigal [32] as part of the DOE-JGI genome annotation pipeline [33], followed by a round of manual curation using the JGI GenePRIMP pipeline [34]. The predicted CDSs were translated and used to search the NCBI non-redundant database, UniProt, TIGR-Fam, Pfam, PRIAM, KEGG, COG, and InterPro database. These data sources were combined to assert a product description for each predicted protein. Additional gene prediction analysis and functional annotation was performed within the IMG-ER platform [35]. Pathway assessment for genomic insights also utilized the ‘MicroScope’ pipeline [36].

## Genome properties

The assembly of the draft genome sequence consists of 22 scaffolds amounting to 3,409,949 bp, and the G + C content is 67.2% (Table 3). Of the 3,092 genes predicted, 3,033 were protein-coding genes, and 59 RNAs; No pseudogenes were identified. The majority of the protein-coding genes (82.0%) were assigned a putative function while the remaining ones were annotated as hypothetical proteins. The distribution of genes into COGs functional categories is presented in Table 4.

**Table 3:** **Genome statistics**

**Attribute**	**Value**	**% of total**
Genome size (bp)	3,409,949	100.00%
DNA coding (bp)	3,058,064	89.68%
DNA G + C (bp)	2,291,117	67.19%
DNA scaffolds	22	
Total genes	3,092	100.00%
Protein-coding genes	3,033	98.09%
RNA genes	59	3.26%
Pseudogenes	0	0%
Genes in internal clusters	Unknown	
Genes with function prediction	2,536	82.02%
Genes assigned to COGs	2,549	82.44%
Genes assigned Pfam domains	2,617	84.67%
Genes with signal peptides	403	13.03%
Genes with transmembrane helices	675	21.83%
CRISPR repeats	4

**Table 4:** **Number of genes associated with the 25 general COG functional categories**

**Code**	**Value**	**% age**	**Description**
J	158	5.64	Translation, ribosomal structure and biogenesis
A	5	0.18	RNA processing and modification
K	147	5.25	Transcription
L	98	3.50	Replication, recombination and repair
B	1	0.04	Chromatin structure and dynamics
D	36	1.28	Cell cycle control, cell division, chromosome partitioning
V	37	1.32	Defense mechanisms
T	93	3.32	Signal transduction mechanisms
M	145	5.17	Cell wall/membrane/envelope biogenesis
N	17	0.61	Cell motility
U	69	2.46	Intracellular trafficking, secretion, and vesicular transport
O	124	4.43	Posttranslational modification, protein turnover, chaperones
C	184	6.57	Energy production and conversion
G	172	6.14	Carbohydrate transport and metabolism
E	325	11.60	Amino acid transport and metabolism
F	68	2.43	Nucleotide transport and metabolism
H	133	4.75	Coenzyme transport and metabolism
I	122	4.35	Lipid transport and metabolism
P	159	5.67	Inorganic ion transport and metabolism
Q	91	3.25	Secondary metabolites biosynthesis, transport and catabolism
R	343	12.24	General function prediction only
S	274	9.78	Function unknown
-	543	17.56	Not in COGs

The total is based on the total number of protein coding genes in the genome.

## Insights from the genome sequence

Analysis of the genome of *M. perideroedes* Gr1^T^ indicates the potential for storage of polyphosphate, PHAs and glycogen, with the former two polymers supported by selective stains in axenic culture and *in situ* strains in activated sludge [1,9]. Storage of such polymers is common in *Bacteria*, and shown to be key to the metabolic strategies of several activated sludge organisms, such as the PAO and GAO phenotypes [37]. The PAO utilize aerobically stored polyphosphate to energize anaerobic carbon uptake. Whilst *Meganema* spp. appear to be able to store polyphosphates, they are unable to assimilate carbon anaerobically [9]. This may in part be due to the absence of the low affinity phosphate *Pit* transport gene, suggested to be key to the use of polyphosphate for energizing anaerobic carbon uptake in the PAO [38,39]. Distribution of *Meganema* spp. appears somewhat restricted to industrial WWTPs, without the anaerobic tanks implemented in EBPR plants. Thus the ability to assimilate PHA likely provides advantage during intermittent periods of carbon starvation or under the unbalanced growth conditions (i.e. high COD to N:P ratio) that often characterize industrial waste streams. Such an explanation was suggested for members of the alphaproteobacterial genus *Amaricoccus*, which also assimilate relatively high PHA reserves and only appear in high abundance in aerated systems treating industrial wastes [40-42].

A novel finding with analysis of the Gr1^T^ genome was the apparent potential for methylotrophic growth. Putative genes for a methanol dehydrogenase (EC. 1.1.2.7), the formaldehyde oxidation pathway (glutathione-dependent), and a formate dehydrogenase (EC 1.2.1.2), were collocated on a putative operon in the genome. These together catalyze the oxidation of methanol to carbon dioxide via formaldehyde [43]. Analysis of potential assimilatory pathways for C1 compounds [43] revealed that key genes were missing for the described serine and ribulose monophosphate pathways, but present for the CBB cycle. Therefore, methanol may be assimilated, via oxidation to CO_2_, through the CBB carbon fixation pathway. Such a phenotype, sometimes referred to as “pseudomethylotrophy” or “autotrophic methylotrophy” [44], has previously been demonstrated for other related members of the order *Rhizobiales* [43,45]. Given the annotated potential for facultative methylotrophy, experimental validation of the ability was assessed in pure culture and for *in situ* community strains present in an environmental sample (for details see Additional file 1). Attempts to grow strain Gr1^T^ on media with methanol as the sole carbon source were unsuccessful. More comprehensive experimental work is required to assess the ability for, and nature of, methylotrophic growth of the Gr1^T^ strain. Methanol assimilation was also not detected for probe-defined *in situ* strains of the genus in the Grindsted WWTP (Additional file 1). The same negative result was obtained for formate assimilation in previous FISH-MAR investigations of the genus [9]. Thus, the ability for methylotrophy is yet to be empirically demonstrated for the genus and the importance of methanol metabolism remains to be resolved. In the case of the activated sludge environment, utilisation of other carbon sources *in situ*, including stored PHA, would be more energetically favorable. Methanol and/or formate oxidation to CO_2_ may supplement energy derived from other sources, or may not be important substrates for these organisms in activated sludge. This is consistent with previous observations that microorganisms in environmental systems are demonstrated to have more specialized physiologies and niches despite metabolic potentials for more diverse activities [46].

Putative denitrification genes were not located in the genome, supporting axenic characterisation of the Gr1 strain, which was unable to grow with nitrate as electron acceptor [1]. *In situ* strains of the genus have been demonstrated to assimilate some substrates anoxically in the presence of nitrate or nitrite, indicating an ability for denitrification [9]. Thus, members of the genus appear to vary in their potential for denitrification. The capacity to fix atmospheric nitrogen is common for other methylotrophic bacteria [43], but the absence of a nitrogenase (EC 1.18.6.1) indicates that this is not the case for *M. perideroedes* Gr1^*T*^.

## Taxonomic proposals

### Description of *Meganemaceae* fam. nov.

*Meganemaceae* (Me.ga.nem.a'ce.ae. N.L. neut. n. *Meganema,* type genus of the family; suff. -*aceae* ending denoting a family; N.L. fem. pl. n. *Meganemaceae,* the family of *Meganema*).

Filamentous morphology with irregular disc shaped cells. Cells stain Gram-negative and, Nile Blue and Neisser positive. The major quinone is Q-10. Fatty acid profiles are dominated by C_18:1_ω7*c*; characteristic hydroxy acids are C_14:0_ 3-OH and C_18:0_ 3-OH. *Meganemaceae* belongs to the order *Rhizobiales* and the type genus is *Meganema*.

## Conclusions

The draft genome sequence of *M. perideroedes* Gr1^T^ is 3.4 Mbp, consisting of 22 scaffolds with 3,033 protein-coding genes. The annotated ability of the organism for facultative methylotrophy could not be demonstrated in either pure culture or for *in situ* strains. Further work is required to elucidate the role of these pathways for the organism, and why they are maintained in the genome. The genome sequence presented here provides a resource for more detailed investigations of this biotechnologically important organism. Based on 16S rRNA gene analysis we formally propose the novel family *Meganemaceae* to include the genus *Meganema*.

## Additional file

Additional file 1:
**Experimental validation of methylotrophy for the Gr1** ^**T**^ **and** ***in situ*** **strains of the genus** ***Meganema.***

## Abbreviations

CBB: Calvin-Bensen-Bassham
COD: Chemical oxygen demand
EBPR: Enhanced biological phosphorus removal
FISH: Fluorescence *in situ* hybridization
GAO: Glycogen accumulating organism
IMG-ER: Integrated Microbial Genomes-Expert Review
MAR: Microautoradiography
MIGS: Minimal information about a genome sequence
PAO: Polyphosphate accumulating organism
PHA: Polyhydroxyalkanoate
WWTP: Wastewater treatment plant
