# Supplementary material for: High quality draft genome sequence of Meganema perideroedes str. Gr1T and a proposal for its reclassification to the family Meganemaceae fam. nov
Source: Stand Genomic Sci. 2015 Feb 27;10:23. doi: 10.1186/s40793-015-0013-1 (PMC4511698; doi:10.1186/s40793-015-0013-1)
Supplement: Additional file 1: — Experimental validation of methylotrophy for the Gr1 T and in situ strains of the genus Meganema. [file 40793_2015_13_MOESM1_ESM.pdf]

Supplementary information: High quality draft genome sequence of *Meganema perideroedes* str. Gr1^T

Simon J. McIlroy, Alla Lapidus, Trine R. Thomsen, James Han, Matthew Haynes, Elizabeth Lobos, Marcel Huntemann, Amrita Pati, Natalia N. Ivanova, Victor Markowitz, Susanne Verbarg, Tanja Woyke, Hans-Peter Klenk, Nikos Kyrpides, and Per H. Nielsen

Experimental validation of methylotrophy

Given the annotated potential of the *M. perideroedes* Gr1^T strain for facultative methylotrophy, experimental validation of the ability was assessed in pure culture and for *in situ* community strains present in an environmental sample.

Experimental procedures

Growth conditions for axenic culture experiments

M. perideroedes Gr1^T was grown on R2A [1] at 25 or 30 °C. In order to determine the essential components of R2A for growth, variations of the media were tested, with the exclusion of one or more of the included carbon sources for each combination (i.e. glucose, pyruvate, peptone, casamino acids and yeast extract). All combinations were supplemented with 1 g l^{-1} of ammonium chloride as a nitrogen source. Also tested was the "*Methylobacterium* media", used for methylotrophic growth of the related *Methylobacterium* genus [2], supplemented with a defined complex mixture of vitamins [3].

FISH-MAR analysis of *in situ* strains of *Meganema*

The FISH-MAR method was essentially the same as described elsewhere [4, 5]. Briefly, 2 ml aliquots of mixed activated sludge (1gSS/L), sampled from the Grindsted WWTP December 2013, were incubated for 3 h with 10 µCi H^3-labelled methanol (American Radiolabeled Chemical Inc., Bio Nuclear AB, Sweden) with addition of up to 2 mM unlabelled methanol under aerobic conditions. The incubations were conducted in 9 ml serum bottles closed with thick butyl rubber stoppers. Following incubation the cells were immediately fixed with freshly prepared 4% [w/v] paraformaldehyde as described by Nielsen [6]. Aliquots (30 µl) of the incubated biomass were gently homogenised between glass coverslips. FISH was performed essentially as described by Nielsen [6] with the equimolar amounts of the Meg983 and Meg1028 oligonucleotide probes [7], and the EUBmix probe set [8, 9], targeting the *Meganema* and most *Bacteria*, respectively. Probes were labelled with either FLUOS [5(6)-carboxyfluorescein-*N*-hydroxy-succinimide ester] or the sulfoindocyanine dye Cy3. Following FISH, slides were coated in Ilford K5D emulsion (Polysciences Europe GmbH, Eppelheim, Germany) and

exposed in the dark for up to 18 days before being developed. Microscopic analysis of the FISH-MAR biomass was performed with an Axioskop epifluorescence microscope (Carl Zeiss, Oberkochen, Germany).

Results

Attempts to grow strain Gr1^T on media with methanol as the sole carbon source were unsuccessful, with the strain requiring an unknown component of yeast extract for growth. When grown on reduced levels of yeast extract (6 mg/l), biomass yield was substantially higher with the addition of glucose (0.5 g/l) but not methanol (1 % [v/v])(data not shown), suggesting that the latter was not utilized for growth. The ”*Methylobacterium* media” did not support growth either, even with the inclusion of vitamins. More comprehensive experimental work is required to assess the ability for, and nature of, methylotrophic growth of the Gr1^T strain. Methanol assimilation was also not detected for probe-defined *in situ* strains of the genus in the Grindsted WWTP (**Figure S1**).

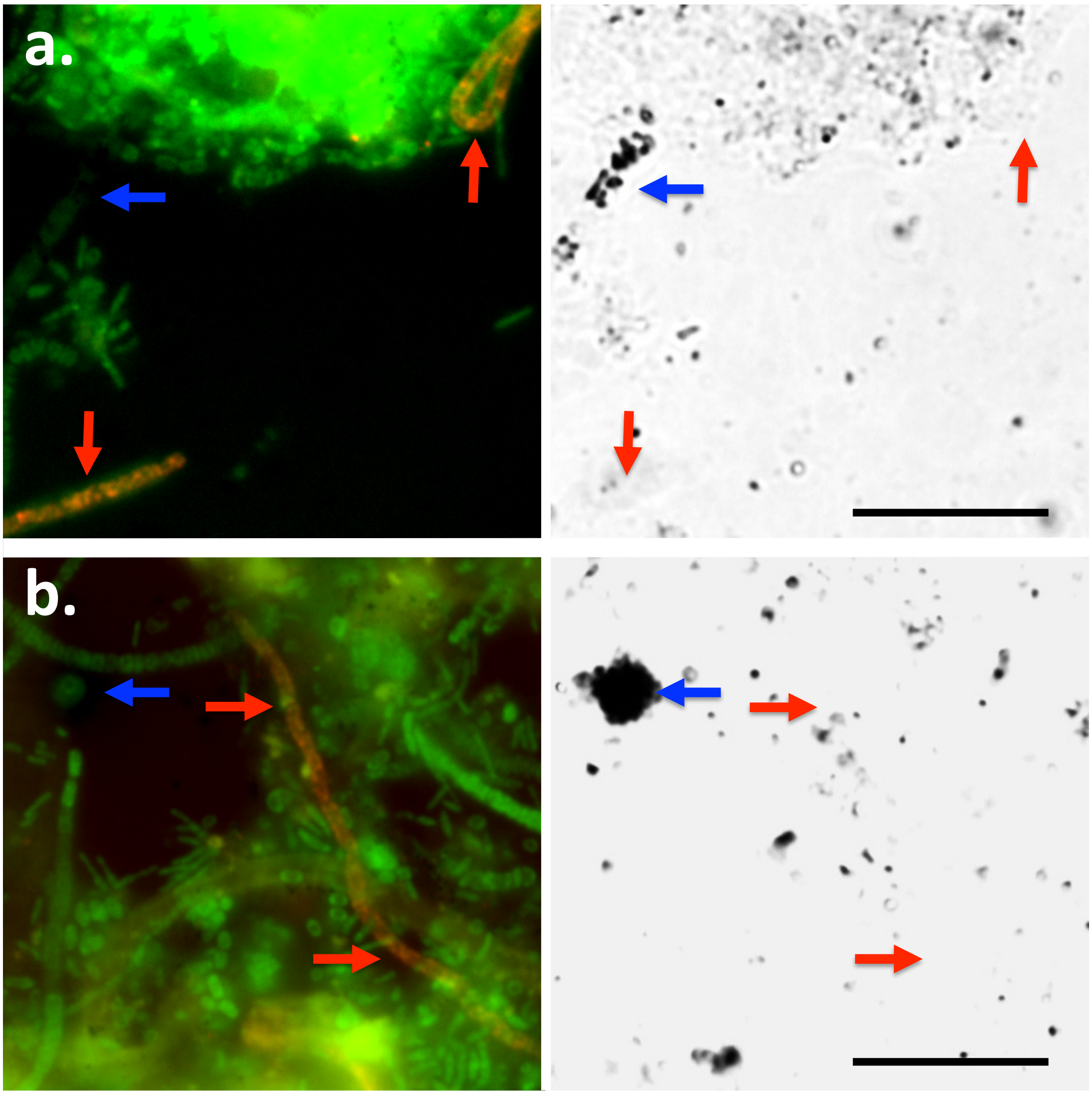

Figure S1. FISH-MAR micrographs of biomass from the Grindsted WWTP incubated with H^3-methanol. **a. and b.** FISH, and corresponding bright field micrograph, with the *Meganema* (Meg983 and Meg1028 probe set [1] Cy3 = red) and all bacteria (EUBMix probe set [8,9] FLUOS = green) (red + green = orange/yellow). Red arrows mark MAR-negative *Meganema* filaments and blue arrows indicate non-target cells with a positive MAR signal (black silver granule deposits). Scale bars represent 10 µm.

References

1. Reasoner DJ, Geldreich EE: **A new medium for the enumeration and subculture of bacteria from potable water.** *Appl Environ Microbiol* 1985; **49:**1-7.

2. Kelly DP, McDonald IR, Wood AP: **Family *Methylobacteriaceae*.** In *The Prokaryotes – Alphaproteobacteria and Betaproteobacteria*. Edited by Rosenberg E, Delong EF, Lory S, Stackebrandt E, Thompson F. Heidelberg: Springer-Verlag; 2014: p. 313-340.

3. Williams TM, Unz RF: **The nutrition of *Thiothrix*, type 021N, *Beggiatoa* and *Leucothrix* strains.** *Water Res* 1989; **23:**15-22.

4. Nielsen JL, Nielsen PH: **Advances in microscopy: microautoradiography of single cells.** *Method Enzymol* 2005; **397:**237-256.

5. Kong Y, Nielsen J, Nielsen P: **Microautoradiographic study of *Rhodocyclus*-related polyphosphate-accumulating bacteria in full-scale enhanced biological phosphorus removal plants.** *Appl Environ Microbiol* 2004; **70:**5383-5390.

6. Nielsen JL: **Protocol for fluorescence *in situ* hybridization (FISH) with rRNA-targeted oligonucleotides.** In *FISH Handbook for Biological Wastewater Treatment.* Edited by Nielsen P, Daims H, Lemmer H. London: IWA Publishing; 2009: 73-84.

7. Thomsen T, Blackall L, de Muro M, Nielsen J, Nielsen P: ***Meganema perideroedes* gen. nov., sp. nov., a filamentous alphaproteobacterium from activated sludge.** *Int J Syst Evol Microbiol* 2006; **56:**1865-1868.

8. Amann RI, Binder BJ, Olson RJ, Chisolm SW, Devereux R, Stahl DA: **Combination of 16S rRNA-targeted oligonucleotide probes with flow cytometry for analyzing mixed microbial populations.** *Appl Environ Microbiol* 1990; **56:**1919-1925.

9. Daims H, Brühl A, Amann R, Schleifer K, Wagner M: **The domain-specific probe EUB338 is insufficient for the detection of all Bacteria: development and evaluation of a more comprehensive probe set.** *Syst Appl Microbiol* 1999; **22:**434-444.
